# miRNA panel from HER2+ and CD24+ plasma extracellular vesicle subpopulations as biomarkers of early-stage breast cancer

**DOI:** 10.1186/s13058-025-02029-2

**Published:** 2025-05-22

**Authors:** Griffin B. Spychalski, Andrew A. Lin, Stephanie J. Yang, Hanfei Shen, Jean Rosario, Kyle Tien, Kate French, Miriyam Ghali, Stephanie Yee, Melinda Yin, Michael D. Feldman, Emily F. Conant, Susan P. Weinstein, Erica L. Carpenter, David Issadore, Anupma Nayak

**Affiliations:** 1 Perelman School of Medicine, University of Pennsylvania, Philadelphia, PA USA; 2 Department of Bioengineering, School of Engineering and Applied Science, University of Pennsylvania, Philadelphia, PA USA; 3 Department of Biology, School of Arts and Sciences, University of Pennsylvania, Philadelphia, PA USA; 4 Division of Hematology-Oncology, Department of Medicine, Perelman School of Medicine, University of Pennsylvania, Philadelphia, PA USA; 5 Department of Pathology and Laboratory Medicine, Perelman School of Medicine, University of Pennsylvania, 3400 Spruce St., Philadelphia, PA 19104 USA; 6 Department of Radiology, Perelman School of Medicine, University of Pennsylvania, Philadelphia, PA USA; 7 Department of Electrical and Systems Engineering, School of Engineering and Applied Science, University of Pennsylvania, Philadelphia, PA USA

**Keywords:** Early detection biomarkers, BI-RADS 4 breast lesions, Extracellular vesicles, Liquid biopsy, MiRNA sequencing

## Abstract

**Background:**

Mammography screening has improved early breast cancer detection, leading to reduced mortality and lower rates of advanced breast cancer. However, mammography has a high false positive rate that results in over a million invasive breast biopsies of benign lesions in the US each year. Therefore, there is a need for noninvasive, blood-based diagnostics that can accurately assess risk of malignancy for women with indeterminate lesions identified by mammography, such as BI-RADS category 4 breast lesions. The aim of this study is to identify biomarkers from multiplexed extracellular vesicle liquid biopsy that can accurately classify mammographically detected BI-RADS 4 lesions.

**Methods:**

We analyzed plasma from 113 prospectively enrolled subjects with BI-RADS 4 breast lesions, including 86 women with benign lesions and 27 women with malignant lesions (including 12 with stage I invasive carcinoma and 14 with ductal carcinoma *in situ*). None of the invasive carcinomas were metastatic. From each plasma sample, we used track etched magnetic nanopore technology to separately isolate HER2 and CD24 expressing extracellular vesicles (EVs) and measured their miRNA cargo using next-generation sequencing. We evaluated the performance of EV-miRNA biomarkers for classifying malignancy and applied LASSO classification to identify a panel of four complementary EV miRNA biomarkers that we validated by qPCR.

**Results:**

We identified 19 differentially enriched miRNA from HER2+ EVs and 11 differentially enriched miRNA from CD24+ EVs of women with malignant lesions compared to benign lesions. We observed individual miRNA with an AUC of up to 0.87 for miR-340-5p from HER2+ EVs and 0.75 for miR-223-3p from CD24+ EVs. LASSO classification selected a panel of four complementary EV miRNA for classifying breast cancer: miR-340-5p (HER2+ EVs), miR-598-3p (CD24+), miR-15b-5p (HER2+), and miR-126-3p (CD24+).

**Conclusions:**

HER2+ and CD24+ EV subpopulations contain complementary biomarkers suitable for validation in larger studies that can accurately detect early-stage breast cancer among women with BI-RADS category 4 breast lesions.

**Supplementary Information:**

The online version contains supplementary material available at 10.1186/s13058-025-02029-2.

## Background

Breast cancer is the leading cause of cancer in women in the United States and is the second leading cause of cancer-related deaths [1]. Mammography screening has greatly improved early breast cancer detection, resulting in a 41% drop in 10-year mortality risk and a 25% decrease in the rate of advanced breast cancer cases [2]. Despite enormous investments and progress in improving image quality, mammographic screening suffers from a high false positive rate (61% after 10 yrs. of annual screening starting at age 40), and results in over one million breast biopsies on women whose lesions are found to be benign in the United States each year [2]. After breast imaging - including mammogram, breast ultrasound, and/or breast MRI - the likelihood of having a malignant tumor is scored using the Breast Imaging Reporting and Data System (BI-RADS) categories. The likelihood of malignancy of BI-RADS category 5 lesions is >95%. However, the likelihood of malignancy for subjects classified as having BI-RADS category 4 lesions is broad, ranging between >2% and ≤95%, and therefore, the American College of Radiology mandates these subjects have a diagnostic biopsy [3]. Although breast core needle biopsy is deemed 'minimally invasive,' it is expensive, time-intensive, and may lead to anxiety, pain, and inconvenience for patients, while contributing an additional $3.07 billion to US annual healthcare spending [4]. Therefore, there is a need for developing a cost-effective and accessible blood-based diagnostic test that can more accurately classify BI-RADS 4 breast lesions as either benign or malignant. If successful, this technology could reduce the number of unwarranted tissue biopsies without reducing the clinical sensitivity of breast imaging.

Liquid biopsy, which aims to measure tumor-derived material released into biofluids, has widely recognized potential to replace or supplement invasive tissue biopsies [5, 6]. However, the clinical translation of liquid biopsy biomarkers for breast cancer diagnosis - including circulating tumor cells (CTCs) and cell-free DNA (cfDNA) – has been limited in successful detection of early stage cancers [7–11]. Heterogeneity of biomarker expression across cancers, and within non-cancer patients has led to poor sensitivity and specificity [7–11]. Extracellular vesicles are nanoscale particles shed by numerous cell types into biofluids, and they contain multiple molecular cargoes which reflect their cell origin. Since they are released by viable tumor cells across tumor stages, they have enhanced sensitivity and specificity for detecting early-stage disease [5, 6, 12, 13]. Previous studies have shown that selectively isolating tumor-associated EV subpopulations based on surface protein expression can improve the sensitivity and specificity of EV biomarkers, providing significant potential to improve cancer diagnoses [14–17]. In our previous work, we have engineered an immunomagnetic nanofluidic chip - Track Etched Magnetic Nanopore (TENPO) sorting - to specifically enrich EV subpopulations from plasma, analyze their cargo, and identify EV biomarkers for clinical applications [17–21]. This technology has been applied to diagnose and guide the treatment of pancreatic cancer, profile traumatic brain injuries, and predict neurologic prognosis following cardiac arrest [17–21]. TENPO sorting overcomes prior limitations of EV subpopulation isolation techniques by enabling precise fluid control across millions of magnetic nanopores for higher throughput and robust operation directly on plasma [17, 18].

In this manuscript, we build on the previous work on the nanomagnetic isolation of EVs and develop a panel of breast cancer-associated EV miRNA biomarkers for classifying the malignancy of BI-RADS 4 breast lesions. To address the heterogeneity of tumor phenotype between patients and the heterogeneous EVs that are emitted from each cell, we apply the specificity of TENPO to separately isolate either HER2+ or CD24+ EV sub-populations and perform a combined analysis of the biomarkers packaged within these complementary sets of EVs. Previous studies have reported that HER2+ EVs are a promising diagnostic for HER2+ breast cancer, and they have been applied to predict patient response to trastuzumab therapy [22–24]. Furthermore, there is increasing interest in the analysis and targeted treatment of HER2-low breast cancers that express HER2 (HER2 immunohistochemistry 1+ or 2+, but FISH negative) because of the apparent success of HER2-targeted treatment for HER2-low breast cancers [25]. Recent studies have investigated the role of breast cancer-derived extracellular vesicles as diagnostics of HER2-low breast cancer to supplement immunohistochemistry [26]. However, the variable expression of HER2 across breast cancers could limit the clinical sensitivity and specificity of HER2+ EVs as individual biomarkers [27]. Therefore, we elected to separately survey CD24+ EVs as a complementary subpopulation due to previous reports of CD24+ EVs as a serum biomarker for breast cancer [28]. Moreover, CD24 has been found to be expressed in approximately 85% of invasive breast carcinomas, providing a robustly expressed target for breast cancer-derived EVs [29]. In this work, we found that by isolating EVs enriched from CD24+ and HER2+ EV sub-types, we can gain a more comprehensive view of a developing cancer than is possible with either single EV sub-population, and thus make more accurate clinical classifications. Specifically, from a prospective cohort of women with BI-RADS 4 breast lesions, we used TENPO to isolate both HER2+ EVs and CD24+ EVs and then performed next generation miRNA sequencing on each isolate to identify EV miRNA biomarkers that distinguish cancer versus noncancer patient samples. In a set of plasma from patients with malignant BI-RADS 4 breast lesions (*N* = 27) compared to EVs isolated from patients with benign BI-RADS 4 breast lesions (*N* = 86), we found 20 differentially enriched miRNAs in the HER2+ EV isolate and 11 in the CD24+ EV isolate. Only 6 of these miRNAs were shared as differentially enriched biomarkers between the HER2+ and CD24+ EVs, suggesting that these EV subpopulations represent complementary biomarkers for the classification of BI-RADS 4 breast lesions. We used LASSO classification to identify a panel of four EV miRNA that classified the cohort of BI-RADS 4 breast lesions with an ensemble model accuracy of 0.88. Finally, we validated our miRNA panel by qPCR, laying the groundwork for validation of these discovered biomarkers in a larger, multi-center prospective clinical study. Taken together, this study reports a dual-EV subpopulation isolation approach to identify new biomarkers for the classification of early-stage breast cancer among women with BI-RADS 4 breast lesions.

## Methods

### Patient sample collection and processing

**Fig. 1:**
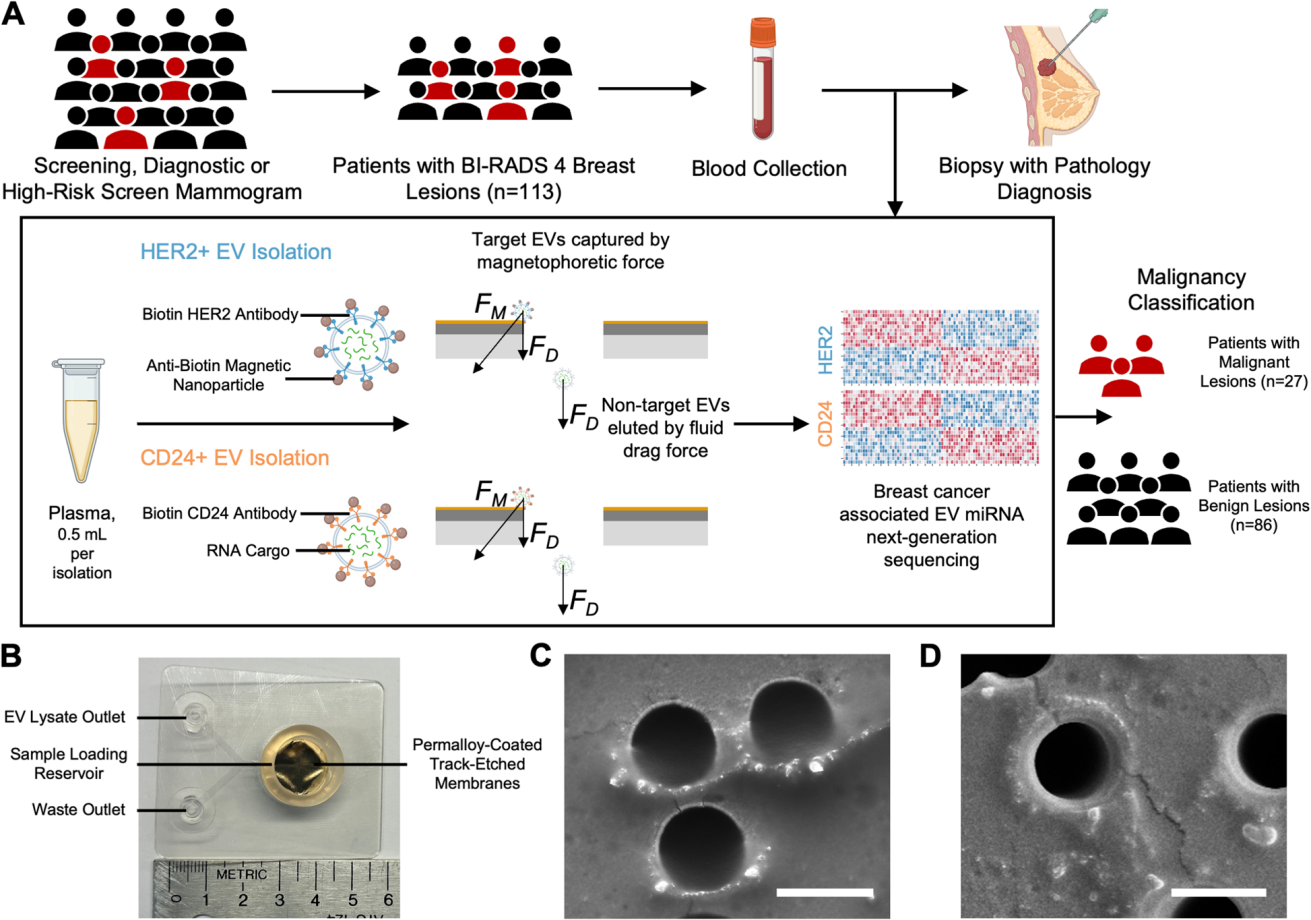
Combining miRNA biomarkers from HER2+ and CD24+ EV subpopulations for diagnosis of early-stage breast cancer. **A** The study population consists of 113 women with BI-RADS category 4 breast lesions, including 27 women with malignant breast lesions and 86 women with benign breast lesions. We immunomagnetically label EVs targeting HER2 or CD24 and then isolate the EV subpopulations using TENPO devices. By sequencing the EVs’ miRNA cargo, we identify biomarkers to accurately classify malignancy. **B** The TENPO device. **C** Scanning electron micrograph of HER2+ EVs isolated on TENPO surface. **D** CD24+ EVs isolated on TENPO surface. Scale bars are 3 μm

A total of 200 women assigned with BI-RADS 4 breast lesions on mammogram between November 2019 and May 2021 were prospectively enrolled in the study after providing signed informed consent at the Hospital of the University of Pennsylvania (Philadelphia, PA) under IRB Protocol #833588. The study was conducted in accordance with the Declaration of Helsinki. The inclusion criteria included women aged 18 years or older who were undergoing screening or diagnostic breast imaging at the Hospital of the University of Pennsylvania, had a BI-RADS category 4 final assessment, and were able to provide informed consent. Individuals with a previous history of cancer or who were pregnant were excluded. BI-RADS categories were scored by breast radiologists according to the American College of Radiology’s criteria [3]. Whole blood was collected from participants before the core biopsy procedure on the day of the scheduled biopsy (Fig. 1A). Venous blood was collected in K_2_EDTA vacutainers (Becton Dickinson) and processed to plasma within 3 hours after blood draw, as described previously [18]. Plasma was aliquoted and stored at −80°C up to 4 years until analysis.

Of the 200 consented participants, whole blood samples could not be collected from 22 participants (Supplementary Fig. S1). For an additional 22 participants, core biopsy was canceled due to an inability to visualize the imaging target at the time of biopsy procedure. One case was excluded after chart review upon discovering that a recent core biopsy procedure was performed prior to the blood sample collection, which could have confounded our liquid biopsy analysis.

The ground truth pathology diagnosis on core biopsy was established by a pathologist specializing in breast pathology. The final pathology result at excision was used as the ground truth when available; one participant was reclassified from atypical ductal hyperplasia at core biopsy to ductal carcinoma in situ (DCIS) at final excision. Fine needle aspiration was performed in place of a core biopsy for two cases. Following pathology diagnosis, there were 27 participants with a malignant breast lesion, including 13 women with invasive carcinomas and 14 women with DCIS (Table 1). Of the 13 women with invasive carcinomas, 12 had stage I carcinomas and 1 had stage III carcinoma (Table 2). Two women with invasive carcinoma were identified to have HER2+ cancer, indicating overexpression of HER2 (Table 2). However, 8 out of 13 women were found to have detectable HER2 expression with HER2 immunohistochemistry (IHC) scores greater than or equal to 1 (Table 2). 123 women were identified with a benign breast lesion, including 10 women with high-risk benign lesions, including intraductal papilloma (*N* = 7), radial scar (*N* = 1), and atypical ductal hyperplasia (*N* = 2), which did not demonstrate malignancy at surgery (Table 1).

**Table 1:** Clinical characteristics of study population

	**Age range** **(median)**	**Race,** **Ethnicity**	**Gender**	**BI-RADS Category**	**Final Pathology Diagnosis**	**Type of Mammography**
Benign (*n = 86*)	27–84 (48)	*n* = 4 Asian *n* = 38 Black *n* = 43 White *n* = 1 Not Provided *n* = 3 Hispanic *n* = 84 Non-Hispanic	*n* = 86 Female *n* = 0 Male	*n* = 37 4A *n* = 34 4B *n* = 13 4C *n* = 1 4A + 4B *n* = 1 4B + 4C	*n* = 18 Fibrocystic change *n* = 18 Fibroadenoma *n* = 12 Benign breast tissue *n* = 9 Mixed benign pathology *n* = 6 Cystic duct ectasia *n* = 7 Intraductal papilloma *n* = 4 Cystic papillary apocrine metaplasia *n* = 3 Reactive lymph node *n* = 2 Atypical ductal hyperplasia *n* = 1 Amyloidosis *n* = 1 Benign cyst *n* = 1 Fat necrosis *n* = 1 Hamartoma *n* = 1 Hemangioma *n* = 1 Lactating adenoma *n* = 1 Radial scar	*n* = 44 Screening *n* = 10 High risk screening *n* = 32 Diagnostic
Malignant (*n = 27*)	40 - 79 (60)	*n* = 0 Asian *n* = 18 Black *n* = 9 White *n* = 0 Hispanic *n* = 27 Non-Hispanic	*n* = 27 Female *n* = 0 Male	*n* = 3 4A *n* = 8 4B *n* = 15 4C *n* = 1 4A + 4C	*n* = 11 Invasive ductal carcinoma, NOS *n* = 1 Invasive lobular carcinoma *n* = 1 Mixed, invasive ductal/lobular carcinoma *n* = 14 Ductal carcinoma *in situ*	*n* = 21 Screening *n* = 1 High risk screening *n* = 5 Diagnostic

**Table 2:** Final pathology classifications of malignant BI-RADS 4 lesions

	Biomarker Subtype		TNM stage			Grade	
Malignant (n = 27)	Invasive Carcinoma n = 8 ER+ PR+ HER2- n = 1 ER- PR- HER2+ n = 3 ER- PR- HER2- n = 1 ER+ PR+ HER2+ HER2 IHC n = 2 Score 3 n = 1 Score 2 n = 5 Score 1 n = 5 Score 0	DCIS n = 13 ER+ PR+ n = 1 ER+ PR-	T stage n = 14 Tis n = 1 T1 mic n = 4 T1a n = 2 T1b n = 5 T1c n = 1 T3	N stage n = 24 N0 n = 1 N0(i+) n = 1 N1 mic n = 1 N1a	M stage n = 27 M0 n = 0 M1	Invasive Carcinoma n = 3 Grade 1 n = 3 Grade 2 n = 7 Grade 3	DCIS n = 0 Low n = 12 Int n = 2 High

For the EV analysis, we analyzed plasma samples from all 27 participants with malignant breast lesions. However, we selected 86 of the 128 women with benign breast lesions to be included in the study; there were no statistically significant differences in the demographics and pathologies between the analyzed subset and the full cohort by a Chi-squared test (Supplementary Table S1). Electronic medical records were re-reviewed in September 2023 (2+ years post-blood draw) to verify that no participant in the benign cohort received a cancer diagnosis following blood collection. We followed STARD 2015 guidelines for reporting diagnostic accuracy studies [30].

### Sample size calculation and power analysis

The necessary sample size was calculated using the ssizeRNA package in R [31]. The total miRNA (with non-zero counts) was estimated at 1000, and the proportion of non-differentially expressed miRNAs was estimated at 0.95 with a mean read count of 10, a dispersity of 0.1, and an average fold change of 2 among differentially expressed miRNAs. To achieve 80% power at an FDR-corrected α of 0.05, a sample size of at least 20 was required (Supplementary Fig. S2).

### Breast cancer cell culture

The breast cancer cell lines BT-474, MDA-MB-453, and MDA-MB-2321 were purchased from ATCC and cultured in RPMI 1640 (BT-474) or Leibovitz’s L-15 (MDA-MB-453 and MDA-MB-231) with 10% fetal bovine serum, 1% L-glutamine, and 1% penicillin/streptomycin in a 37 °C incubator with 5% CO_2_. Cells were routinely tested for mycoplasma with the MycoAlert kit (Lonza #LT07-318). To produce conditioned medium, cells were seeded in their corresponding medium with 10% exosome-depleted FBS at 1.3 × 10^7^ cells/dish in 15 cm tissue culture dishes and cultured for 5 days. The conditioned medium was collected and centrifuged at 1600×g for 10 minutes, then 3000×g for 10 minutes in a room temperature swinging-bucket centrifuge to remove cellular debris. The conditioned media was stored at −80°C in 1 mL aliquots.

### Nanoparticle tracking analysis

To quantify EV concentration in conditioned cell culture media, we isolated EVs from cell culture media with total exosome isolation reagent (from cell culture media) (Invitrogen). The samples were serially diluted in DI water and measured by a ZetaView PMX220 Twin at the Extracellular Vesicle Core at the University of Pennsylvania. A particle cutoff of 45 to 255 nm was used to estimate EV concentration.

### EV surface marker ELISA

We identified protein surface markers for immunomagnetically labeling breast cancer-derived EVs using a whole-EV ELISA with breast cancer cell conditioned culture media. Anti-human CD9, CD63, and CD81 capture antibodies (BioLegend) were added to wells of a high-binding 96-well plate (Greiner) at a concentration of 2 μg mL^−1^ (100 μL) in alkaline coating buffer (30 mM Na_2_CO_3_ (Sigma-Aldrich) and 70 mM NaHCO_3_ (Sigma-Aldrich) in DI water) and allowed to coat overnight at 4 °C on a nutating mixer. Subsequently, 100 μL of isolated BT-474, MDA-MB-453, or MDA-MB-231 breast cancer EV samples (10^8^ EVs, concentration measured by nanoparticle tracking analysis), prepared with total exosome isolation reagent (from cell culture media) (Invitrogen) and resuspended in alkaline coating buffer, were added to each well and incubated overnight at 4 °C on a nutating mixer. Next, each well was blocked with 200 μL SuperBlock (in PBS) blocking buffer (ThermoFisher) with 1% bovine serum albumin (BSA; Sigma-Aldrich) overnight at 4 °C on a nutating mixer. Biotinylated anti-human detection antibodies — CD9 (BioLegend), CD63 (BioLegend), CD81 (BioLegend), CD24 (BioLegend), EpCAM (BioLegend), ErbB2 (HER-2; Novus Biologicals), CD156c (ADAM10; BioLegend), BRST-2 (GCDFP-15; BioLegend), and mammaglobin A (3 C8; Invitrogen) — and corresponding isotype biotinylated antibodies — mouse IgG1 κ (BioLegend), mouse IgG2a κ (BioLegend), mouse IgG2b κ (BioLegend) — were individually added to corresponding wells at a concentration of 2 μg mL^−1^ (100 μL) and incubated for 1 hour at room temperature. Then, 100 μL of HRP-streptavidin (ThermoFisher), diluted 1:16,000 in SuperBlock blocking buffer with 1% BSA, was added to each well and incubated for 15 minutes at room temperature. Kinetic fluorescent measurements were conducted with a plate reader (Infinite M PLEX, TECAN) and the fluorescence rate of change was calculated for each well over 3 minutes while the rate of change remained linear. SuperBlock T20 (TBS) blocking buffer was used as a washing buffer between steps.

### Scanning electron microscopy

Scanning electron microscope (SEM) imaging was performed at the Cell and Developmental Biology Microscopy Core (Perelman School of Medicine, University of Pennsylvania) following a protocol previously reported by our group [17, 32]. After we isolated EVs on TENPO devices, the captured EVs were immobilized with 50 mM Na-cacodylate buffer (500 μL, three washes) and fixed for 2 hours with 2% glutaraldehyde in 50 mM Na-cacodylate buffer. Next, the EV-coated permalloy membranes were excised from the devices with a razor. The samples were dehydrated in graded ethanol concentrations through 100% over 1.5 hours; dehydration in 100% ethanol was completed three times. Dehydrated samples were incubated in 50% HMDS in ethanol for 20 minutes and then washed with 100% HMDS (Sigma-Aldrich) three times and air-dried overnight. The samples were mounted and sputter coated with gold palladium. Samples were imaged using a Quanta 250 FEG scanning electron microscope (FEI, Hillsboro, OR, USA) at 10 kV accelerating voltage [33].

### Validation of EV subpopulation isolation by plasma spike-in experiment

We validated the sensitivity and specificity of our immunomagnetic isolation of breast cancer-derived EVs using a plasma spike-in model. Conditioned culture media from BT-474, MDA-MB-453, or MDA-MB-231 breast cancer cells (0.125 mL, 1.6 × 10^8^ EVs) was spiked into a cocktail of plasma isolated from 3 patients with benign BI-RADS 4 breast lesions (0.5 mL) and diluted to 1 mL in 1xDPBS (Gibco) to replicate a malignant sample. Non-conditioned clean culture media (media not cultured with cancer cells; 0.125 mL) was added to the benign plasma cocktail (0.5 mL) and diluted to 1 mL in 1xDPBS (Gibco) to replicate a benign sample. Samples were passed through a 0.22 μm PES filter (CellTreat) to remove debris prior to immunomagnetic labeling.

### Tumor-derived EV miRNA isolation by TENPO

EVs from patient’s K_2_EDTA-collected plasma (0.5 mL per isolation) were diluted to 1 mL in 1xDPBS (Gibco), passed through a 0.22 μm PES filter (CellTreat), and immunomagnetically labeled using biotinylated antibodies and anti-biotin ultrapure magnetic nanoparticles (50 nm diameter; Miltenyi Biotec). Antibodies used in this study included biotin anti-human CD24 (BioLegend) and biotin anti-human ErbB2 (HER-2) monoclonal antibody (Novus Biologicals). For either a CD24 or HER-2 EV isolation, the corresponding biotinylated antibody was added to the diluted sample to achieve a final concentration of 5 μg mL^−1^ and incubated for 1 hour at room temperature on a nutating mixer. Then, anti-biotin magnetic nanoparticles (50 μL; Miltenyi Biotec) were added to the samples and incubated for 1 hour at room temperature on the nutating mixer. The labeled plasma samples were loaded into the reservoir of the TENPO device (Chip Diagnostics) and connected to a programmable syringe pump (Braintree Scientific) to provide negative pressure to pull the sample through the device at a flow rate of 1.5 mL h^−1^.

Details on the design and fabrication of TENPO have been reported previously [17, 18]. A permanent magnet (NdFeB disc magnet, *d* = 1.5 inches, *h* = 0.75 inches; K&J Magnetics) was placed beneath the TENPO device to magnetize TENPO’s paramagnetic Ni_80_Fe_20_ film (200 nm) and the superparamagnetic-labeled EVs. TENPO is designed such that, as the sample is pulled through the device, EVs that are sufficiently labeled by magnetic nanoparticles are specifically captured at the edge of the pores, while the vast background of EVs flow through the 3 μm diameter pores and can be discarded [17]. The captured EVs were lysed on the chip in QIAzol lysis reagent (700 μL; Qiagen), incubated for 3 minutes, and then collected as lysate. RNA was extracted from the lysate off-chip (miRNeasy Serum/Plasma Advanced Kit, Qiagen). EV miRNA was eluted in 20 μL RNase-free water and stored at −80°C or immediately used for further analysis.

For TENPO-isolated EV protein analysis, immunomagnetically captured EVs were released in Pierce IgG Elution Buffer (500 μL; Thermo Scientific), incubated for 5 minutes, and then collected as eluate. The EV eluate was analyzed following the EV surface marker ELISA method described previously.

### EV miRNA sequencing and statistical analysis

QIAseq miRNA Library Kit (96 indices, compatible with Illumina platforms; Qiagen) was used to prepare a library from isolated EV miRNA. Library concentration was calculated prior to sequencing by Qubit high sensitivity dsDNA quantification assay kit (Thermo Fisher). PhiX was spiked into the library prior to sequencing at 1% concentration (PhiX Control v3; Illumina). The library was sequenced using a NovaSeq 6000 S1 kit with 75 bp single-read for 100 cycles (Illumina, Next-Generation Sequencing Core, University of Pennsylvania, Philadelphia, PA). Expression quantification was performed using the Qiagen RNA-seq Analysis Portal (Qiagen) by aligning to the miRBase v22 reference library. Expression counts were normalized by RUVSeq [34] and DESeq2 [35] to minimize technical bias between samples from batch library preparation.

Differentially enriched EV miRNA between malignant and benign samples were identified using DESeq2 with the Wald test for statistical significance and Benjamini-Hochberg false discovery rate correction [35]. Area under the receiver-operator characteristic curve (AUC) was calculated for each miRNA with its 95% confidence interval using the pROC package in R [36] and tested for statistically significant differences in AUCs between miRNA using DeLong’s method in R [37]. Correlation between differentially enriched miRNA was quantified as the Kendall tau correlation coefficient calculated using the SciPy package in Python. Pathway analysis of differentially enriched miRNA was performed using the DIANA-miRPath (v4.0) application using KEGG terms/pathways [38].

### Selection of EV miRNA panel

To identify potential EV miRNA candidates for breast cancer diagnosis, we applied the feature selection algorithm least absolute shrinkage and selection operator (LASSO) on EV miRNA sequencing results to find the most informative miRNAs [19, 39]. Prior to LASSO selection, we filtered the miRNA such that the median expression counts for both benign and malignant samples were greater than zero to remove low-abundance miRNA. Due to sample size imbalance between the benign and malignant cohorts, we performed the synthetic minority oversampling technique (SMOTE) to rebalance the cohort size and minimize bias due to overclassification of the majority class (benign cohort) [40]. We evaluated the LASSO-selected markers by generating a learning curve for an ensemble machine learning model’s accuracy as a function of the number of EV miRNA feature inputs. The ensemble model included K-nearest neighbors, support vector machine, linear discriminate analysis, logistic regression, and naive Bayes classifiers averaged as a stacking ensemble to mitigate overfitting [19]; the model was carried out in Python. We selected four miRNAs from the results of the LASSO classification and measured their expression by qPCR to evaluate correlation of sequencing and qPCR data.

### EV miRNA qPCR

The TaqMan advanced miRNA cDNA synthesis kit (Applied Biosystems) was used to convert miRNA to cDNA, and the TaqMan fast advanced master mix for qPCR (Applied Biosystems) was used with corresponding TaqMan advanced miRNA assays (Applied Biosystems). For the cancer cell conditioned media spike-in experiment previously described, TaqMan advanced miRNA assays for hsa-miR-101-3p, hsa-miR-21-5p, and hsa-miR-27a-3p were performed following manufacturer instructions. For the sequencing validation experiment previously described, TaqMan advanced miRNA assays for hsa-miR-340-5p, hsa-miR-598-3p, hsa-miR-15b-5p, and hsa-miR-126-3p were performed following manufacturer instructions. Cycle threshold (Ct) values were measured on a Bio-Rad CFX384 C1000 thermocycler, with thresholds set automatically by the instrument at 10 times the standard deviation of baseline fluorescence.

## Results

### Validation of immunomagnetic isolation of HER2+ EVs and CD24+ EVs

We first used an *in vitro* model to identify surface proteins abundantly expressed on breast cancer derived EVs. We harvested EV conditioned cell culture media from BT-474 invasive ductal carcinoma cells and MDA-MB-453 metastatic breast carcinoma cells and identified HER2 and CD24 expression by whole-EV ELISA (Supplementary Fig. S3 A-C). The BT-474 cell line is known to overexpress HER2 and CD24, while the MDA-MB-453 cell line expresses both HER2 and CD24 at lower levels [41]. In addition, we identified an absence of HER2 and CD24 expression on EVs harvested from the MDA-MB-231 cell line, consistent with its previously reported expression pattern [41]. Next, we validated HER2 and CD24 as immunomagnetic targets for the specific isolation of breast cancer associated EV miRNA. We immunomagnetically isolated HER2+ EVs and CD24+ EVs separately from EV conditioned culture media (1.6×10^8^ EVs, concentration measured by nanoparticle tracking analysis) from the BT-474, MDA-MB-453, or MDA-MB-231 cell lines spiked into 0.125 mL plasma isolated from patients with benign BI-RADS 4 breast lesions (Supplementary Fig. S3D). We measured the EV miRNA yield by qPCR for miRNAs reported in previous studies as differentially expressed in either plasma EVs of breast cancer patients or breast cancer cell culture EVs [42–44]. We report a 4 to 6-fold enrichment of breast cancer associated EV miRNA in both HER2 and CD24 antibody labeled sample compared to isotype antibody immunolabeled samples for the BT-474 and MDA-MB-453 spike-in models (Student’s t-test p < 0.05) (Supplementary Fig. S3E, G). For the MDA-MB-231 spike-in model, there was no difference in EV miRNA yield for either HER2 or CD24 antibody labeled samples compared to isotype labeled samples, consistent with its lack of HER2 or CD24 expression (Supplementary Fig. S3 F, H). Furthermore, we repeated the whole-EV ELISA to characterize protein expression on TENPO-isolated HER2+ and CD24+ EVs and validated expression of CD24 and HER2 (Supplementary Fig. S3E, G).

### Identification of HER2+ EV and CD24+ EV miRNA biomarkers

**Fig. 2:**
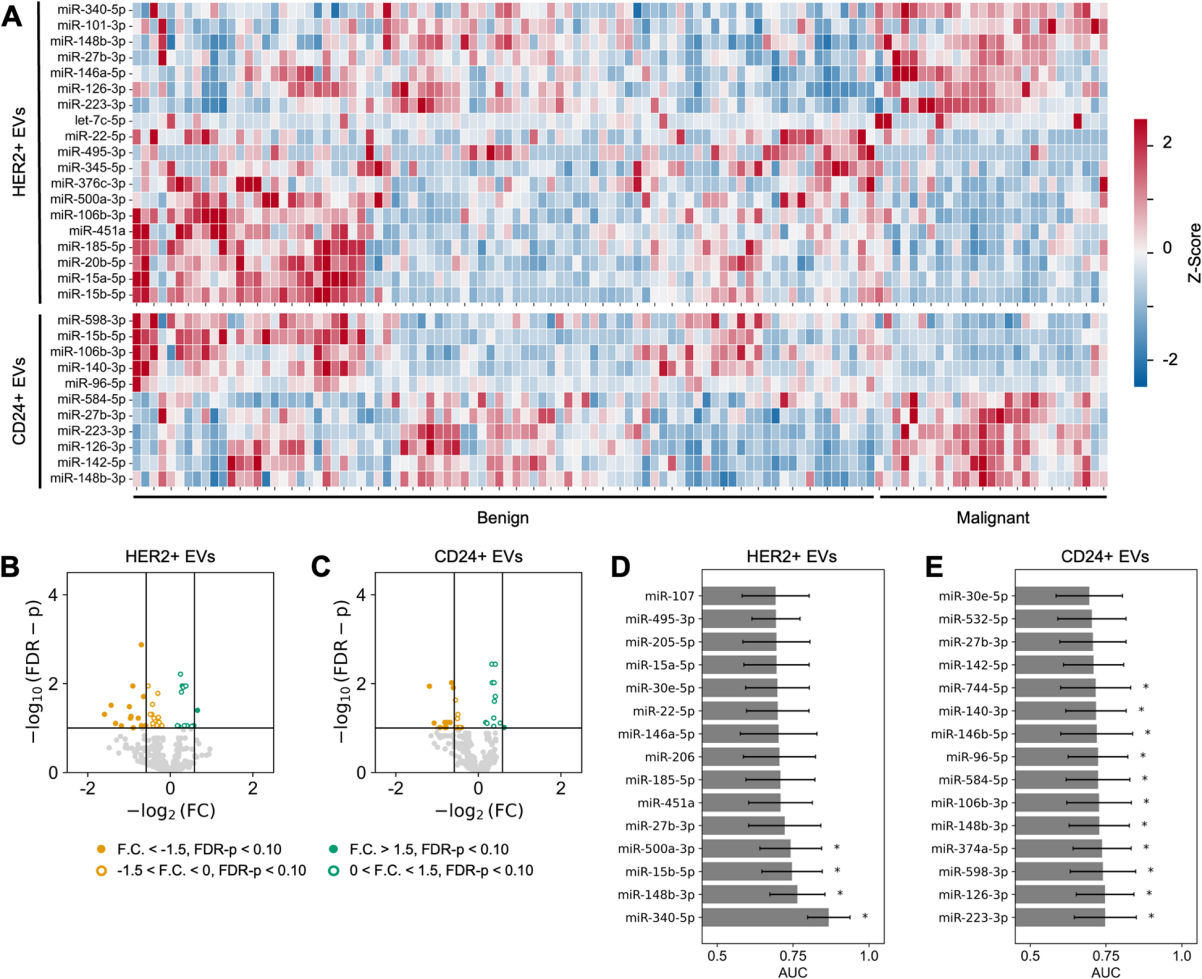
Identification of HER2+ and CD24+ EV-miRNA biomarkers.** A** Heatmap shows Z-score of differentially enriched miRNA biomarkers from HER2+ or CD24+ EVs isolated from each of the benign (n = 86) and malignant (n = 27) plasma samples (FDR-corrected Wald test p < 0.05). **B** Volcano plot of miRNA from HER2+ EVs. **C** Volcano plot of miRNA from CD24+ EVs. **D** Area under the receiver-operator characteristic curve (AUC) for the classification of malignancy by HER2+ EV-miRNA. **E** AUC for the classification of malignancy by CD24+ EV-miRNA (* denotes FDR-corrected Mann-Whitney U-test p < 0.05). Error bars represent the 95% confidence interval generated using DeLong’s method

We identified miRNA biomarkers that were differentially enriched in HER2+ plasma EVs between women with malignant and benign BI-RADS 4 breast lesions using next generation sequencing. Nineteen miRNAs were identified as differentially enriched in HER2+ EVs with Wald test FDR-corrected p-values below 0.05 (Fig. 2A and 2B). Eight miRNAs were upregulated and eleven miRNAs were downregulated in HER2+ EVs from women with malignant lesions relative to women with benign lesions. Two miRNAs had an AUC greater than 0.75 and Mann-Whitney U test FDR-corrected p-value less than 0.05, including miR-340-5p (AUC = 0.87, 95% CI: 0.80–0.94) and miR-148b-3p (AUC = 0.76, 95% CI: 0.67–0.86) (Fig. 2D).

Next, we identified miRNA biomarkers that were differentially enriched in CD24+ EVs in plasma between women with malignant and benign BI-RADS 4 breast lesions. Eleven miRNAs were identified as differentially enriched in CD24+ EVs with Wald test FDR-corrected p-values below 0.05 (Fig. 2A and 2 C). Six miRNAs were upregulated and five miRNAs were downregulated in CD24+ EVs of patients with malignant lesions relative to patients with benign lesions. The greatest AUCs were demonstrated by miR-223-3p (AUC = 0.75, 95% CI 0.65–0.85) and miR-126-3p (AUC = 0.75, 95% CI 0.65–0.84) (Fig. 2E).

We performed a stratified analysis of the differentially enriched EV miRNA by separately comparing the ten women with high-risk benign lesions at final excision – including atypical ductal hyperplasia, intraductal papilloma, and radial scar – to women with low-risk benign lesions and women with malignant lesions. From the nineteen miRNAs that were differentially enriched in HER2+ EVs between women with malignant and benign lesions, three were differentially enriched in HER2+ EVs between women with low-risk benign and high-risk benign lesions with Wald test FDR-corrected p-values below 0.05 (Supplementary Fig. S4 A). Of the eleven miRNAs that were differentially enriched in CD24+ EVs between women with malignant and benign lesions, three were found to be differentially enriched in CD24+ EVs between women with low-risk benign and high-risk benign lesions with Wald test FDR-corrected p-values less than 0.05 (Supplementary Fig. S4 A).

None of the EV miRNAs differentially enriched between women with malignant and benign lesions were found to be differentially enriched between women with ductal carcinoma *in situ* and invasive carcinoma (Supplementary Fig. S5 A).

We compared the differential enrichment of miRNA in HER2+ EVs and CD24+ EVs between women with malignant and benign BI-RADS 4 lesions to previous studies that have reported differential enrichment of EV miRNA from total EV preparations in plasma EVs of women with breast cancer [45–49]. From the set of previously reported total EV miRNA, only miR-142-5p was found to be differentially enriched in CD24+ EVs from our study (Wald test FDR-corrected p-value < 0.05; AUC = 0.71, 95% CI 0.61–0.81) (Supplementary Table S2).

### Comparison of miRNA biomarkers from HER2+ EVs and CD24+ EVs

**Fig. 3:**
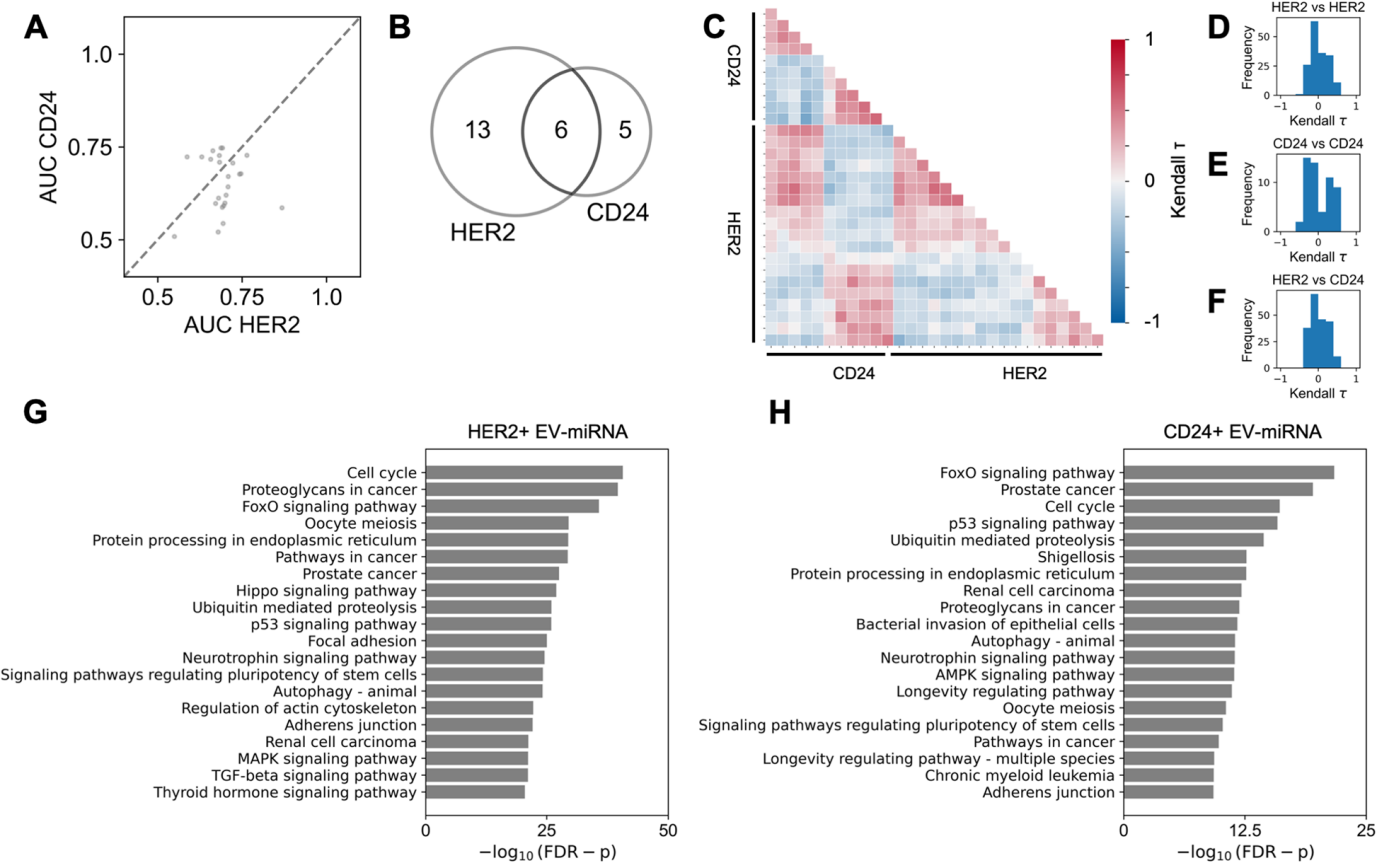
Comparison of EV-miRNA biomarkers from HER2+ and CD24+ EV subpopulations.** A** AUC of differentially enriched EV-miRNA from HER2+ EVs compared to those from CD24+ EVs. **B** Venn diagram of shared and distinct miRNA biomarkers from HER2+ EVs and CD24+ EVs; area is scaled to size of each group. **C** Correlogram of Kendall tau correlation coefficient between differentially enriched EV-miRNA. **D** Relative frequency histogram of Kendall tau correlation coefficient between HER2+ EV miRNAs compared to HER2+ EV miRNAs. **E** Relative frequency histogram of Kendall tau correlation coefficient between CD24+ EV miRNAs compared to CD24+ EV miRNAs. **F** Relative frequency histogram of Kendall tau correlation coefficient between HER2+ EV miRNAs compared to CD24+ EV miRNAs. **G** Enriched pathways associated with differentially enriched HER2+ EV-miRNAs identified by KEGG pathway analysis (FDR-corrected Fisher’s exact test p). **H** Enriched pathways associated with differentially enriched CD24+ EV-miRNAs identified by KEGG pathway analysis

To compare the differentially expressed miRNAs carried by HER2+ EVs and CD24+ EVs, we first evaluated differences in the AUCs of miRNA cargo from each EV subpopulation. We found that of the twenty-four total differentially expressed miRNAs, eight had a significant difference in AUC between the HER2+ and CD24+ EVs (FDR-corrected DeLong’s test p < 0.10). A subset of the miRNAs demonstrated a high AUC (~0.75) for both the HER2+ EVs and CD24+ EVs, suggesting a correlation of expression for those markers (Fig. 3A, B).

Next, we calculated the Kendall tau rank correlation coefficient between each of the differentially expressed EV miRNAs (Fig. 3C). We observed a weak correlation between differentially expressed miRNAs from HER2+ EVs (mean absolute Kendall tau = 0.19) (Fig. 3D), and a slightly stronger correlation between miRNAs from CD24+ EVs (mean absolute Kendall tau = 0.27) (Fig. 3E). We observed a weak correlation between miRNAs from CD24+ EVs compared to miRNAs from HER2+ EVs (mean absolute Kendall tau = 0.20) (Fig. 3F).

We performed a KEGG pathway analysis to identify associations between differentially expressed EV miRNAs and disease processes. Pathway analysis of the differentially enriched HER2+ EV miRNAs (FDR-corrected Wald test p < 0.05) revealed a strong association with various cancers and cancer-associated pathways, including FoxO and p53 signaling, as well as prostate cancer (Fig. 3G). Four miRNAs (miR-27b-3p, miR-126-3p, miR-22-5p, and miR-340-5p) were found to be significantly associated with breast cancer (FDR-corrected Fisher’s exact test p = 2.9×10^−8^). Similarly, pathway analysis of the differentially enriched CD24+ EV miRNAs (FDR-corrected Wald test p < 0.05) revealed strong associations with FoxO and p53 signaling pathways, as well as various cancers (Fig. 3H). Two miRNAs (miR-27b-3p and miR-126-3p) were found to be significantly associated with breast cancer (FDR-corrected Fisher’s exact test p = 4.8×10^−5^).

### Selection of miRNA Biomarker Panel

**Fig. 4:**
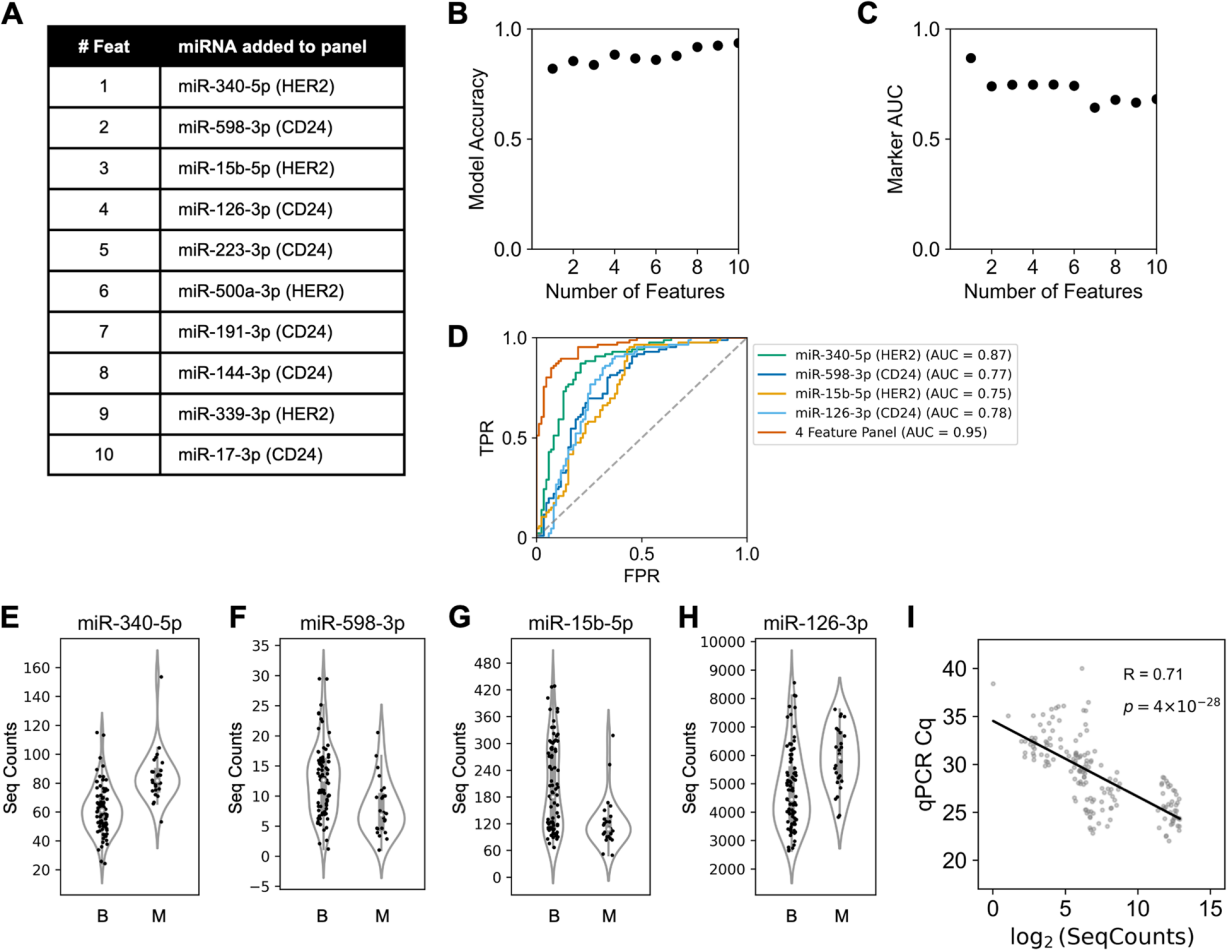
Selection of EV-miRNA biomarker panel.** A** Features selected by LASSO algorithm from either HER2+ or CD24+ EVs, noted in parentheses. As the hyperparameter regulating the cost function is reduced, the panel is expanded to include more features. **B** Learning curve plotting the accuracy of classifying malignancy for an ensemble machine learning algorithm trained using an incremental panel of LASSO-selected features. **C** AUC of individual markers selected by LASSO. **D** AUC ROC curve of true positive rate (TPR) against false positive rate (FPR) for individual miRNA and combined panel. **E-H** Sequencing counts of LASSO-selected features for benign (B) and malignant (M) samples. **I** Correlation of sequencing counts and qPCR values. R denotes Pearson’s R correlation coefficient (two-sided Student’s t-test p-value)

Because individual differentially enriched miRNAs were found to be weakly correlated, we hypothesized that these biomarkers could be algorithmically combined to create a more accurate classifier of malignancy for BI-RADS 4 breast lesions than any individual biomarker [50]. To test this hypothesis, we applied LASSO classification for feature selection of a complementary panel of EV miRNA. We tuned the LASSO hyperparameter, α, to generate panels of varying sizes of EV miRNA biomarkers and calculated the accuracy of each panel for classifying malignancy in an ensemble machine learning model with 5-fold cross validation to generate a learning curve (Fig. 4B).

We identified that an accuracy of 0.88 was achieved with a panel of four weakly correlated EV miRNA: miR-340-5p (HER2+ EVs), miR-598-3p (CD24+ EVs), miR-15b-5p (HER2+ EVs), and miR-126-3p (CD24+ EVs) (Fig. 4E-H). Across panel sizes of four and seven features, the model’s accuracy plateaued at 0.88 (Fig. 4B). Moreover, panels beyond 6 features began to incorporate weakly predictive EV-miRNA with AUC less than 0.70 (Fig. 4C). Therefore, the optimal panel size of four EV-miRNA was selected to maximize model accuracy while minimizing the risk of overfitting from low AUC features. The selected miRNA panel had an AUC of 0.95, along with an optimal sensitivity of 0.87 and a specificity of 0.91 (Fig. 4D).

Additionally, we tested the performance of the miRNA panel for classifying low-risk benign samples compared to the combined group of high-risk benign and malignant samples (Supplementary Fig. S4B-E). The panel had an AUC of 0.93, along with a sensitivity of 0.89 and a specificity of 0.88 (Supplementary Fig. S4 F). There were no statistically significant differences for the miRNA panel for ductal carcinoma *in situ* compared to invasive carcinoma (Supplementary Fig. S5B-E).

Notably, miRNAs from both HER2+ EVs and CD24+ EVs were alternately selected, suggesting complementary information is contained in the EV subpopulations. Publications reporting roles for these EV-associated miRNA in breast cancer and other solid cancers can be found in Supplementary Table S3. We used the TISSUES 2.0 database to compare tissue specificity for each miRNA; the database reports transcriptomics data to reveal gene-tissue associations in human samples [51]. We found that the four selected miRNA are known to be enriched in blood (z-scores greater than 3.2), while miR-340-5p is also known to be enriched in breast cancer cells (z-score = 3.2) (Supplementary Table S3) [51]. To validate the results from our sequencing analysis, we measured the miRNA concentration for each LASSO-selected marker by qPCR (Supplementary Fig. S6 A-D). We observed a strong correlation between sequencing counts and qPCR Cq values for each miRNA (Pearson’s R correlation coefficient: 0.71) (Fig. 4I).

## Discussion

In this study, we developed an extracellular vesicle liquid biopsy to identify miRNA biomarkers packaged within HER2+ and CD24+ plasma EVs for the classification of malignancy in women with BI-RADS 4 breast lesions. We found 24 individual miRNAs that had significant predictive value to classify women with breast cancer, including miR-340-5p from HER2+ EVs with an AUC of 0.87 and miR-223-3p from CD24+ EVs with an AUC of 0.75. It is important to note that 26 out of 27 malignant samples included in this study were diagnosed with Stage 0 or Stage I breast cancer and none exhibited metastatic disease, demonstrating the potential for our assay to accurately detect early-stage breast cancer. Finally, we report a panel of four complementary EV miRNA biomarkers for the classification of BI-RADS 4 breast lesions, and we validated the correlation of our biomarker panel sequencing results with qPCR.

Plasma EV miRNAs were separately isolated from HER2+ EVs or CD24+ EVs in this study with the hypothesis that they would contain independent data to classify malignancy. We are motivated to identify lists of predictive, uncorrelated markers, as they can be fed into machine learning algorithms to achieve classification performance superior to any of the individual features [52, 53]. To evaluate the suitability of our data for incorporation into a machine learning algorithm, we applied LASSO feature selection to our data and scanned LASSO’s hyperparameter to generate a complementary panel of EV miRNA biomarkers. We found that a panel of four EV miRNA biomarkers achieved the greatest accuracy while minimizing the number of biomarkers, which can guard against overfitting. Interestingly, the panel selected EV miRNA biomarkers derived from both HER2+ EVs and CD24+ EVs. HER2 and CD24 were chosen as complementary, rather than mutually exclusive biomarkers for breast cancer EVs. These findings suggest that each EV subpopulation carries miRNA biomarkers, which together offer the highest diagnostic accuracy. We observed a moderately-strong correlation between the sequencing data and qPCR Cq values with Pearson’s R correlation coefficient of 0.71, indicating that these biomarkers can be translated from sequencing to qPCR for clinical formats.

Due to the increased risk of DCIS and invasive breast carcinoma among women with high-risk benign lesions, accurate detection and proper clinical management of high-risk benign lesions is critical. When stratified to compare EV miRNA from women with high-risk benign lesions compared to women with low-risk benign lesions, we observed that several of the differentially enriched EV miRNA were also differentially expressed between women with low-risk benign and high-risk benign lesions. From the panel of four EV miRNA biomarkers, miR-598-3p in CD24+ EVs and miR-15b-5p in HER2+ EVs were differentially enriched between women with high-risk benign lesions compared to women with low-risk benign lesions, and the panel achieved an AUC of 0.93 for classifying women with low-risk benign lesions compared to high-risk benign and malignant lesions. Although follow-up with a larger cohort of women with high-risk benign lesions is necessary, these findings suggest that our assay may accurately distinguish low-risk benign lesions compared to high-risk benign and malignant lesions, in concordance with the necessary clinical management pathways for each lesion.

Our work is primarily distinguished from previous studies by our measurement and analysis of the differential expression of miRNA cargo from two complementary plasma EV subpopulations to identify breast cancer biomarkers. Prior research has largely focused on total EV preparations or single subpopulations [24, 28, 42, 44–49, 54, 55]. Recently, Hu *et al*. isolated EPCAM+ and FAPα+ plasma EVs with a microfluidic immunoaffinity array for breast cancer subtype profiling [56]. In contrast, we have identified miRNA biomarkers from HER2+ and CD24+ plasma EV subpopulations for the classification of early-stage breast cancer.

Our reported EV-based biomarkers have potential to stratify the risk of malignancy for BI-RADS 4 breast lesions, enabling more precise detection and management of early-stage breast cancer, relative to standard mammography, using easily accessible blood samples, including subjects with precancerous lesions within a cohort of subjects with BI-RADS 4 breast lesions. Women with BI-RADS 4 breast lesions included in this study were enriched for Stage 0 and Stage 1 breast cancers, which have failed to be detected with high AUC by previously reported liquid biopsy biomarkers [7, 9, 10, 43, 54]. Here, we report a panel of four complementary biomarkers with individual AUCs up to 0.87 for classifying early-stage breast cancer among BI-RADS 4 breast lesions.

There are several important limitations of our study. First, proteomic analysis of EVs isolated in the HER2+ and CD24+ EV isolates would enable more precise comparison of the two subpopulations of breast cancer-associated EVs. Specifically, single EV analysis techniques could resolve heterogeneity of protein expression between EVs to reveal the distribution of HER2 and CD24 expression between EVs enriched in both HER2+ and CD24+ fractions [57–59]. Moreover, HER2 and CD24 are both expressed in normal tissues and other cancers, including ovarian and gastric cancers, which could limit the clinical specificity of HER2+ and CD24+ EVs as biomarkers of breast cancer [27, 60]. However, our approach to analyze the miRNA cargo of HER2+ and CD24+ can provide additional specificity to improve the performance of our assay compared to measuring absolute levels of each EV population alone. In addition, the biomarkers in our panel are principally tumor-associated, and this assay would likely benefit from combining EV biomarkers from complementary EV subpopulations, such as tumor- and immune-associated EVs, or with additional liquid biopsy biomarkers, such as cfDNA or plasma protein assays. Finally, a larger cohort that includes an independent, prospective validation cohort and multi-center enrollment would further support the clinical utility of the biomarkers reported in this study.

## Conclusions

In summary, we report a biomarker panel composed of miRNA isolated from breast cancer-associated EVs that can differentiate malignant lesions in women diagnosed with BI-RADS 4 breast lesions. By comparing the miRNA biomarkers enriched from both HER2+ and CD24+ EV subpopulations, we have found that the dual isolation of each EV subpopulation provides unique, complementary information to the imaging and classification of BI-RADS 4 breast lesions. Future work can evaluate this noninvasive blood-based assay in a larger cohort and with EVs from multiple cell types (breast cancer cells, immune cells) to further evaluate the performance of our assay in the clinical classification of BI-RADS 4 breast lesions.

## Supplementary Information

Supplementary Material 1.

## Data Availability

The datasets used and/or analyzed during the current study are available from the corresponding author on reasonable request.

## Abbreviations

EV: Extracellular vesicle
BI-RADS: Breast Imaging-Reporting and Data System
CTC: Circulating tumor cell
cfDNA: cell-free DNA
TENPO: Track-etched magnetic nanopore device
DCIS: Ductal carcinoma in situ
BSA: Bovine serum albumin
SEM: Scanning electron microscope
AUC: Area under the receiver-operator characteristic curve
LASSO: Least absolute shrinkage and selection operator
Ct: Cycle threshold
FDR: False discovery rate
TPR: True positive rate
FPR: False positive rate
