# Supplementary material for: miRNA panel from HER2+ and CD24+ plasma extracellular vesicle subpopulations as biomarkers of early-stage breast cancer
Source: Breast Cancer Res. 2025 May 22;27:90. doi: 10.1186/s13058-025-02029-2 (PMC12096773; doi:10.1186/s13058-025-02029-2)
Supplement: Supplementary file 1 — Supplementary Material 1. [file 13058_2025_2029_MOESM1_ESM.docx]

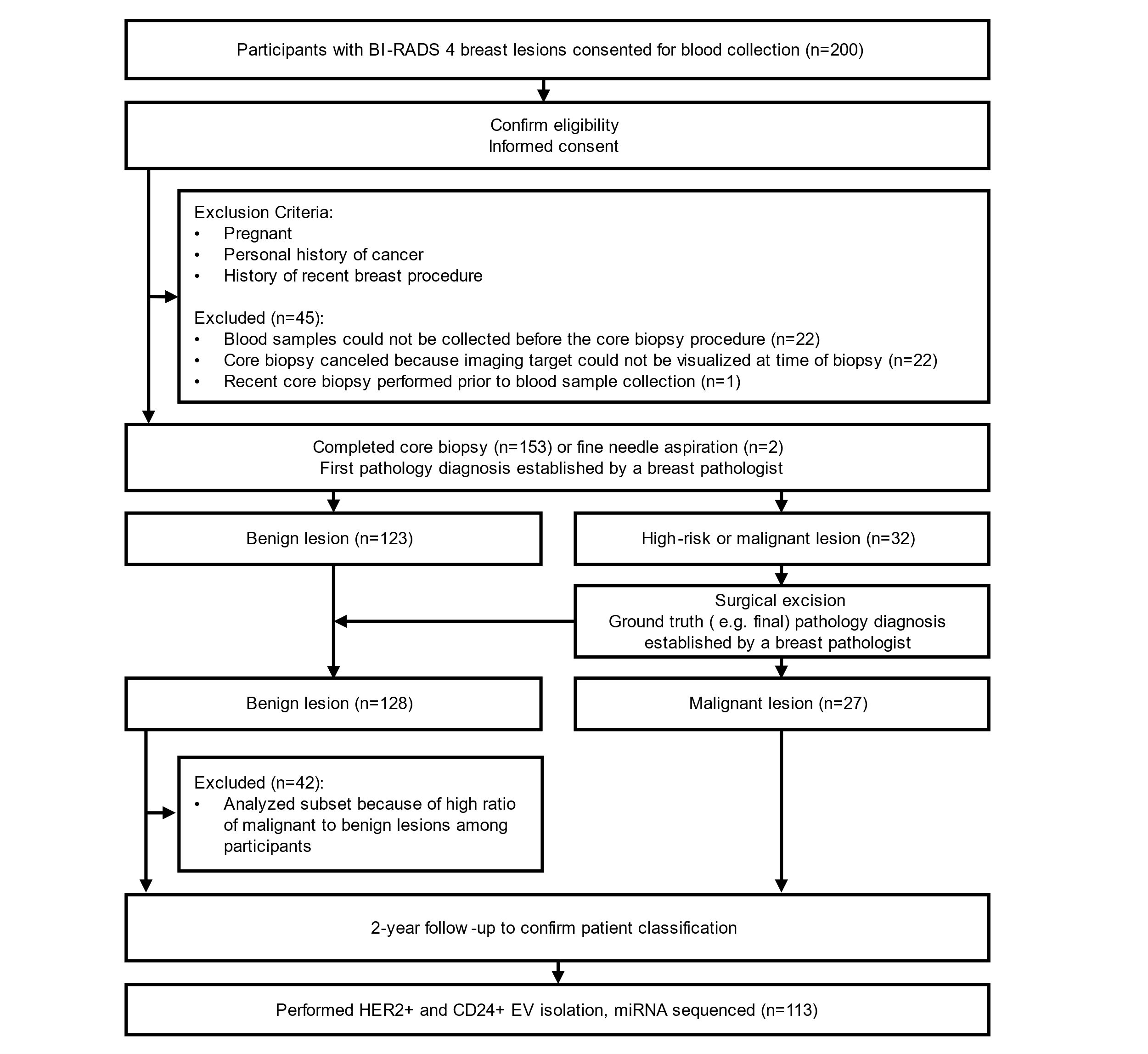


**Supplementary Figure S1.**

Flow chart of study cohort and rationale for participant exclusion.

**Supplementary Table S1.** Clinical characteristics of analyzed study cohort and full study cohort.

|  | **Age range**  **(median)** | **Race,**  **Ethnicity** | **Gender** | **BI-RADS Category** | **Pathology Diagnosis** |
| --- | --- | --- | --- | --- | --- |
| Benign,  Analyzed Subset  (*n* = 86) | 27 – 84 (48) | *n* = 4 Asian (5%)  *n* = 38 Black (44%)  *n* = 43 White (50%)  *n* = 1 Not Provided (1%)  *n* = 3 Hispanic (3%)  *n* = 83 Non-Hispanic (87%) | *n* = 86 Female (100%)  *n* = 0 Male (0%) | *n* = 37 4A (43%)  *n* = 34 4B (40%)  *n* = 13 4C (15%)  *n* = 1 4A + 4B (1%)  *n* = 1 4B + 4C (1%) | *n* = 18 Fibrocystic change (21%)  *n* = 18 Fibroadenoma (21%)  *n* = 12 Benign breast tissue (14%)  *n* = 9 Mixed benign pathology (10%)  *n* = 7 Intraductal papilloma (8%)  *n* = 6 Cystic duct ectasia (7%)  *n* = 4 Cystic papillary apocrine metaplasia (5%)  *n* = 3 Reactive lymph node (3%)  *n* = 2 Atypical ductal hyperplasia (2%)  *n* = 1 Amyloidosis (1%)  *n* = 1 Benign cyst (1%)  *n* = 1 Fat necrosis (1%)  *n* = 1 Hamartoma (1%)  *n* = 1 Hemangioma (1%)  *n* = 1 Lactating adenoma (1%)  *n* = 1 Radial scar (1%)  *n* = 0 Fine needle aspirate, benign (0%)  *n* = 0 Tubular adenoma (0%) |
| Benign,  All Participants  (*n* = 128) | 20 - 84 (47) | *n* = 6 Asian (5%)  *n* = 57 Black (45%)  *n* = 60 White (47%)  *n* = 5 Not Provided (4%)  *n* = 5 Hispanic (4%)  *n* = 123 Non-Hispanic (96%) | *n* = 128 Female (100%)  *n* = 0 Male (0%) | *n* = 58 4A (45%)  *n* = 51 4B (40%)  *n* = 16 4C (13%)  *n* = 2 4A + 4B (2%)  *n =* 1 4B + 4C (1%) | *n* = 23 Fibrocystic change (18%)  *n* = 35 Fibroadenoma (27%)  *n* = 15 Benign breast tissue (12%)  *n* = 14 Mixed benign pathology (11%)  *n* = 9 Intraductal papilloma (7%)  *n* = 7 Cystic duct ectasia (5%)  *n* = 4 Cystic papillary apocrine metaplasia (3%)  *n* = 6 Reactive lymph node (5%)  *n* = 5 Atypical ductal hyperplasia (4%)  *n* = 1 Amyloidosis (1%)  *n* = 1 Benign cyst (1%)  *n* = 2 Fat necrosis (2%)  *n* = 1 Hamartoma (1%)  *n* = 1 Hemangioma (1%)  *n* = 1 Lactating adenoma (1%)  *n* = 1 Radial scar (1%)  *n* = 2 Fine needle aspirate, benign (2%)  *n* = 1 Tubular adenoma (1%) |


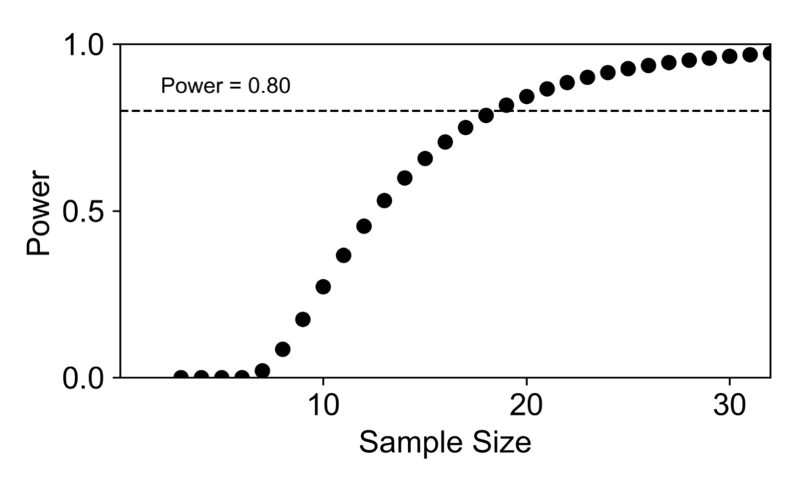


**Supplementary Figure S2.**

Power analysis used to estimate sample size requirements with ssizeRNA package in R. This calculation assumes a total of 1000 miRNA with non-zero counts and a proportion of non-differentially expressed miRNA of 0.95 with an average fold change of 2 among differentially expressed miRNAs.

**
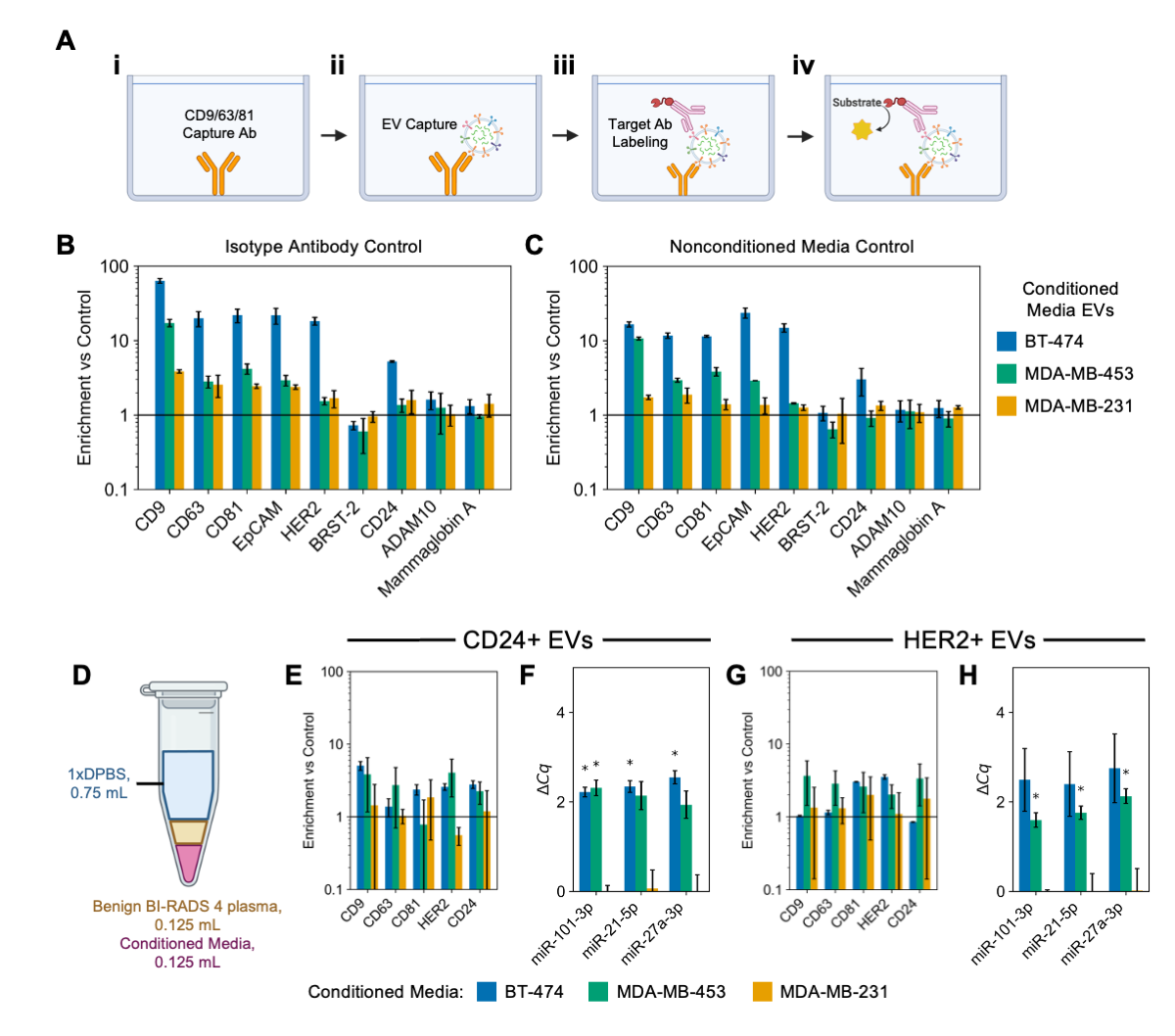
**

**Supplementary Figure S3.**

Development and validation of anti-HER2 and anti-CD24 immunomagnetic pulldowns. **A,** Whole-EV sandwich ELISA workflow to detect surface proteins on breast cancer cell derived EVs. Well plates coated with tetraspanin antibodies captured EVs from breast cancer cell conditioned media, then labeled with biotinylated target antibodies and reacted with HRP-streptavidin. After conversion of HRP substrate, fluorescence is proportional to amount of target surface protein. **B,** Ratio of relative fluorescence unit rate of change for target antibodies relative to isotype antibody controls for EVs from media conditioned by BT-474 (blue), MDA-MB-453 (green), or MDA-MB-231 (red) breast cancer cells. **C,** Ratio of relative fluorescence unit rate of change for conditioned media EVs vs RPMI non-conditioned media control. **D,** Anti-HER2 and anti-CD24 immunomagnetic pulldowns were validated using a cancer spike-in model sample consisting of conditioned media from BT-474, MDA-MB-453, or MDA-MB-231 cells spiked into plasma from patients with benign BI-RADS 4 lesions. **E,** Whole-EV sandwich ELISA to detect surface proteins on CD24+ EVs isolated from cancer spike-in model. **F,** Difference in qPCR Cq values for breast cancer EV-associated miRNAs for anti-CD24 immunomagnetic pulldown relative to isotype antibody control. **G,** Whole-EV sandwich ELISA to detect surface proteins on HER2+ EVs isolated from cancer spike-in model. **F,** Difference in qPCR Cq values for breast cancer EV-associated miRNAs for anti-HER2 immunomagnetic pulldown relative to isotype antibody control. All error bars represent standard error of the mean. * denotes one-sided Student’s t-test p < 0.05.

**
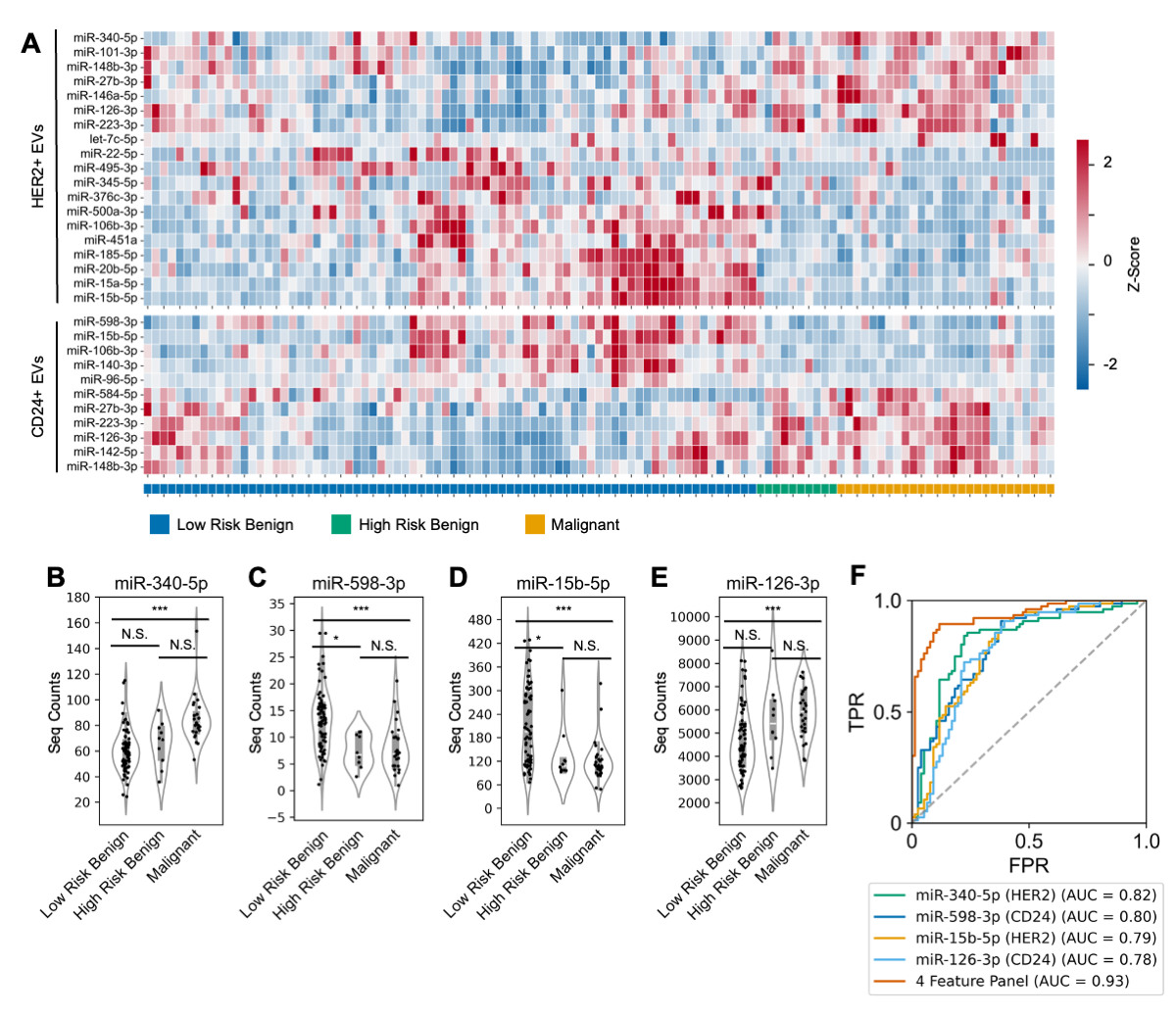
**

**Supplementary Figure S4.**

Identification of HER2+ and CD24+ EV-miRNA biomarkers between low-risk benign, high-risk benign, and malignant pathologies. **A.** Heatmap shows Z-score of differentially enriched miRNA biomarkers from HER2+ or CD24+ EVs isolated from each of low-risk benign (n = 76), high-risk benign (n = 10), and malignant (n = 27) plasma samples (FDR-corrected Wald test p < 0.05). **B-E.** Sequencing counts of LASSO-selected features stratified for low-risk benign, high-risk benign, and malignant samples (* denotes Wald test FDR-corrected p-value < 0.05; *** < 0.001). **F.** AUC ROC curve for individual miRNA and panel for the classification of low-risk benign compared to combined high-risk benign and malignant samples.

**
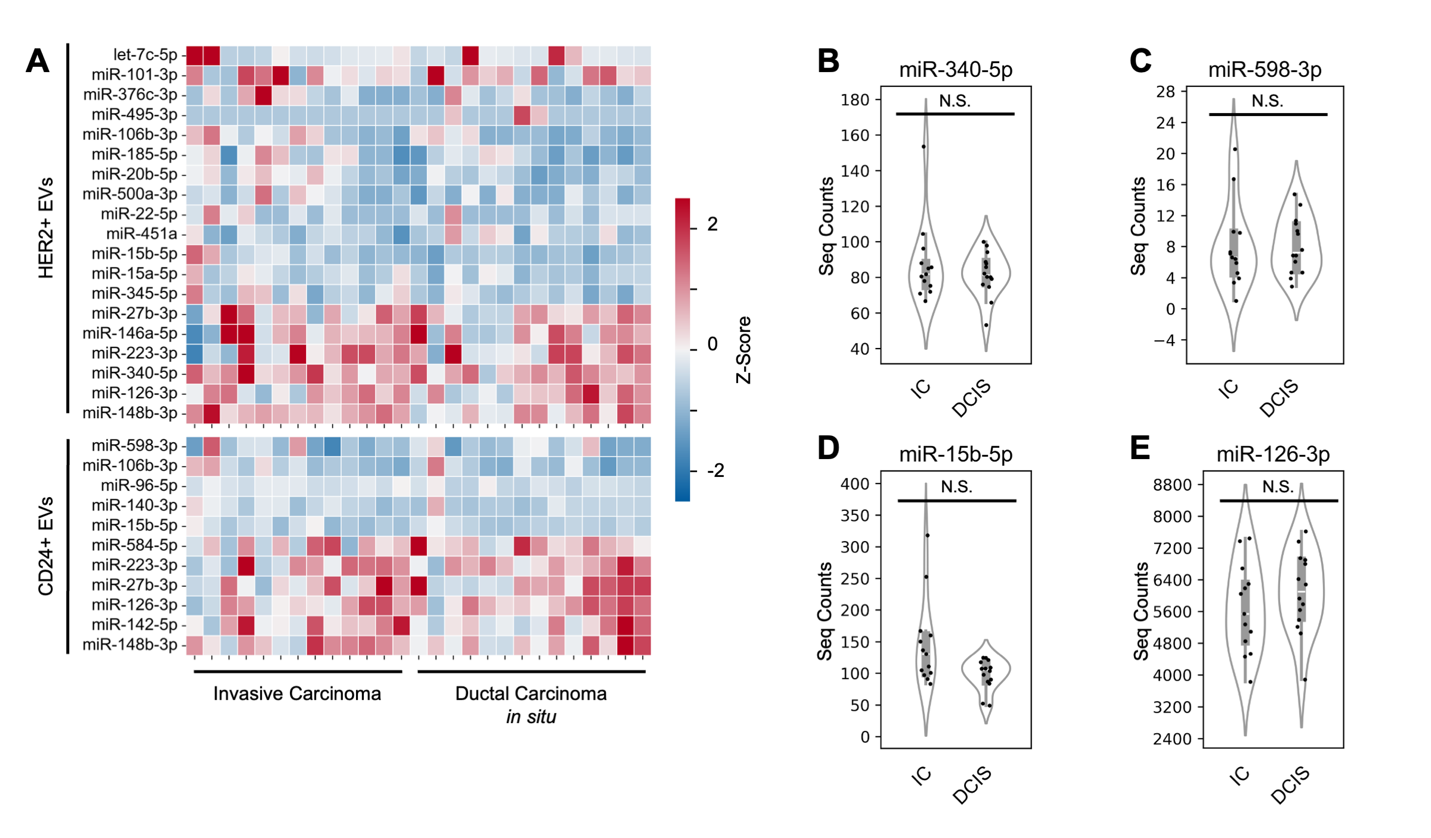
**

**Supplementary Figure S5.**

Identification of HER2+ and CD24+ EV-miRNA biomarkers between invasive carcinoma and ductal carcinoma *in situ* pathologies. **A.** Heatmap shows Z-score of miRNA biomarkers from HER2+ or CD24+ EVs differentially enriched between benign and malignant samples, sorted to show expression for invasive carcinoma (n = 13) and ductal carcinoma *in situ* (n = 14) samples. **B-E.** Sequencing counts of LASSO-selected features stratified for invasive carcinoma (IC) and ductal carcinoma *in situ* (DCIS) samples. N.S. denotes Wald test FDR-corrected p-value > 0.05.

**Supplementary Table S2.** Comparison of previously reported miRNA from total EV preparations for breast cancer diagnosis to enrichment of miRNA in HER2+ and CD24+ EVs (1-5).

|  |  |  |  |  |  |  | **HER2+ EVs** | | **CD24+ EVs** | |
| --- | --- | --- | --- | --- | --- | --- | --- | --- | --- | --- |
| **Reference** | **Breast Cancer Type, Control** | **EV Source, Preparation** | **miRNA** | **log_2_FC**  **(+/-)** | **AUC** | **miRNA Strand** | **log_2_FC** | **AUC**  **(95% CI)** | **log_2_FC** | **AUC**  **(95% CI)** |
| Yan et al. *Molecular Medicine Reports* (2019) | Stage I  (*n* = 12)  Fibroadenoma, mammary adenosis  (*n* = 10) | Plasma  ExoQuick precipitation (Systems Biosciences) | miR-548b-5p | -1.20 | 0.79 |  | 0.00 | 0.51  (0.40-0.61) | 0.68 | 0.55  (0.45-0.65) |
|  |  |  | miR-375 | 1.17 | 0.59 |  | -0.56 | 0.56  (0.44-0.68) | -0.99 | 0.55  (0.44-0.65) |
|  |  |  | miR-655-3p | -1.95 | 0.72 |  | 0.39 | 0.51  (0.43-0.60) | -0.30 | 0.54  (0.46-0.61) |
|  |  |  | miR-24-2-5p | 2.42 | 0.63 |  | 0.02 | 0.51  (0.47-0.55) | Not detected | |
| Kim et al. *Cancer Science* (2021) | Early stage  (*n* = 55)  Locally advanced  (*n* = 7)  Healthy  (*n* = 20) | Plasma  Immunoaffinity (CD49f+ and EpCAM+) | miR-9 | + | 0.71 | miR-9-3p | 0.43 | 0.55  (0.44-0.67) | 0.41 | 0.52  (0.40-0.63) |
|  |  |  |  |  |  | miR-9-5p | -0.07 | 0.53  (0.40-0.66) | 0.13 | 0.56  (0.44-0.68) |
|  |  |  | miR-16 | + | 0.85 | miR-16-1-3p | -0.16 | 0.51  (0.40-0.62) | -0.3 | 0.58  (0.48-0.68) |
|  |  |  |  |  |  | miR-16-2-3p | -0.17 | 0.55 (0.43-0.68) | -0.1 | 0.59  (0.46-0.72) |
|  |  |  |  |  |  | miR-16-5p | 0.02 | 0.52  (0.39-0.65) | 0.11 | 0.53  (0.40-0.66) |
|  |  |  | miR-21 | + | 0.69 | miR-21-3p | -0.60 | 0.67  (0.56-0.78) | -0.46 | 0.61  (0.50-0.73) |
|  |  |  |  |  |  | miR-21-5p | 0.16 | 0.59  (0.46-0.71) | -0.03 | 0.55  (0.44-0.67) |
|  |  |  | miR-429 | + | 0.71 |  | 0.59 | 0.59  (0.47-0.71) | 0.57 | 0.54  (0.42-0.67) |
| Hannafon et al. *Breast Cancer Research* (2016) | Stage 0 to III  (*n* = 16)  Healthy  (*n* = 16) | Plasma  ExoQuick precipitation (Systems Biosciences) | miR-1246 | + | 0.69 |  | -0.28 | 0.59  (0.47-0.72) | 0.12 | 0.52  (0.40-0.64) |
|  |  |  | miR-21 | + | 0.69 | miR-21-3p | -0.60 | 0.67  (0.56-0.78) | -0.46 | 0.61  (0.50-0.73) |
|  |  |  |  |  |  | miR-21-5p | 0.16 | 0.59  (0.46-0.71) | -0.03 | 0.55  (0.44-0.67) |
| Ozawa et al. *Biomolecules* (2020) | Luminal A  (*n* = 16)  Triple-negative  (*n* = 15)  Healthy  (*n* = 16) | Serum  Total exosome isolation reagent (Invitrogen) | miR-320a | + | 0.81 | miR-320a-3p | -0.25 | 0.64  (0.52-0.76) | -0.29 | 0.60  (0.49-0.70) |
|  |  |  |  |  |  | miR-32a-3p | 0.05 | 0.52  (0.48-0.55) | -0.07 | 0.51  (0.49-0.52) |
|  |  |  | miR-142-5p | + | 0.80 |  | 0.27 | 0.68  (0.58-0.79) | 0.41* | 0.71  (0.61-0.81) |
|  |  |  | miR-4433b-5p | + | 0.80 |  | -0.13 | 0.52  (0.39-0.65) | 0.06 | 0.57  (0.45-0.69) |
| Koi et al. *Cancer Science* (2020) | Stage 0 to III  (*n* = 39)  Cancer-free women  (*n* = 36) | Serum  Total exosome isolation reagent (Invitrogen) | miR-21-5p  (IsomiR, 3’ addition C) | 2.05 | 0.69 acc. |  | 0.16 | 0.59  (0.46-0.71) | -0.03 | 0.55  (0.44-0.67) |
|  |  |  | miR-23a-3p | 2.48 | 0.89 acc. |  | -0.20 | 0.64  (0.51-0.77) | 0.08 | 0.58  (0.46-0.70) |

**Supplementary Table S3.** LASSO-selected miRNA panel with relative tissue expression data from the TISSUES 2.0 database (6), along with relevant liquid biopsy literature (7-11).

| **Marker** | **Tissue Expression**  **(Z-score)** | **Relevant Liquid Biopsy-Related Publications** |
| --- | --- | --- |
| hsa-miR-340-5p | - Blood (3.8) - Immune system (3.6) - Lung cancer cell (3.5) - Brain (3.4) - Lung (3.3) - Hepatoma cell (3.3) - Heart (3.3) - Blood vessel (3.2) - Breast cancer cell (3.2) - Breast (3.1) | - Exosomal miR-340-5p was upregulated in the serum of breast cancer patients with recurrence compared to patients without recurrence (Sueta et al, *Oncotarget*, 2017) - Serum exosomal miR-340-5p was significantly correlated with the fraction of Ki67+ tumor cells in breast cancer patients (Curtaz et al, *Int. J. Mol. Sci.*, 2022) |
| hsa-miR-598-3p | - Blood (3.2) - Brain (2.9) - OVCA-4 cell (2.8) - Immune system (2.8) - Saliva (2.7) - Spinal cord (2.6) - Urine (2.6) - Gastric cancer cell (2.6) - Lung cancer cell (2.5) - Colorectal cancer cell (2.4) | - Serum miR-598-3p was significantly downregulated in breast cancer patients relative to healthy subjects. miR-598-3p demonstrated a sensitivity of 95%, a specificity of 85%, and an AUC of 0.94 for detecting breast cancer (Fu et al, *Oncol. Lett.*, 2016) |
| hsa-miR-15b-5p | - Blood (5.2) - Immune system (4.8) - Heart (4.7) - Muscle (4.6) - Brain (4.5) - Blood vessel (4.5) - Liver (4.5) - Lung (4.4) - Urine (4.3) - Intestine (4.2) | - Plasma exosomal miR-15b-5p was significantly downregulated in squamous cell carcinoma patients relative to healthy subjects (Jin et al, *Clin. Cancer Res.*, 2017) |
| hsa-miR-126-3p | - Heart (6.0) - Blood vessel (6.0) - Blood (6.0) - Muscle (5.5) - Immune system (5.4) - HUVEC cell (5.4) - Lung (5.1) - Mesenchymal stem cell (5.1) - Urine (5.0) - Liver (4.9) | - Serum miR-126-3p was significantly downregulated in non-small cell lung cancer patients compared to healthy subjects (Soliman, *Mol. Biol. Rep.*, 2021) |


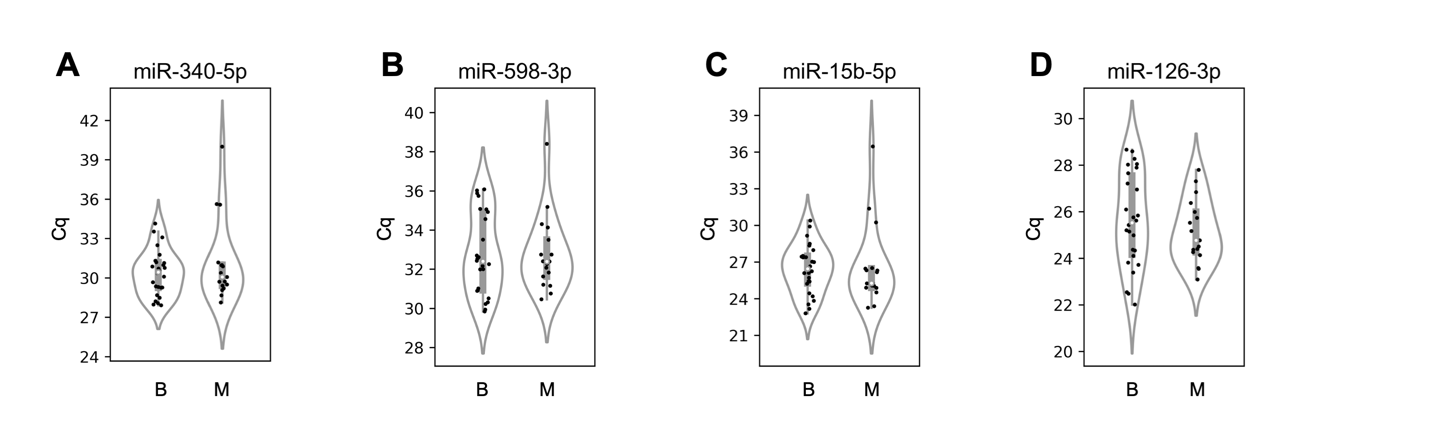


**Supplementary Figure S6.**

Measurement of LASSO-selected EV miRNA by qPCR for benign (B) and malignant (M) samples. **A,** HER2+ EV miR-340-5p. **B,** CD24+ EV miR-598-3p. **C,** HER2+ EV miR-15b-5p. **D,** CD24+ EV miR-126-3p.

**References**

1. Yan C, Hu J, Yang Y, Hu H, Zhou D, Ma M, et al. Plasma extracellular vesicle‑packaged microRNAs as candidate diagnostic biomarkers for early‑stage breast cancer. Molecular Medicine Reports. 2019.

2. Kim MW, Park S, Lee H, Gwak H, Hyun KA, Kim JY, et al. Multi‐miRNA panel of tumor‐derived extracellular vesicles as promising diagnostic biomarkers of early‐stage breast cancer. Cancer Science. 2021;112(12):5078-87.

3. Hannafon BN, Trigoso YD, Calloway CL, Zhao YD, Lum DH, Welm AL, et al. Plasma exosome microRNAs are indicative of breast cancer. Breast Cancer Research. 2016;18(1).

4. Ozawa PMM, Vieira E, Lemos DS, Souza ILM, Zanata SM, Pankievicz VC, et al. Identification of miRNAs Enriched in Extracellular Vesicles Derived from Serum Samples of Breast Cancer Patients. Biomolecules. 2020;10(1):150.

5. Koi Y, Tsutani Y, Nishiyama Y, Ueda D, Ibuki Y, Sasada S, et al. Predicting the presence of breast cancer using circulating small RNAs, including those in the extracellular vesicles. Cancer Science. 2020;111(6):2104-15.

6. Palasca O, Santos A, Stolte C, Gorodkin J, Jensen LJ. TISSUES 2.0: an integrative web resource on mammalian tissue expression. Database. 2018/01/01;2018.

7. Sueta A, Yamamoto Y, Tomiguchi M, Takeshita T, Yamamoto-Ibusuki M, Iwase H. Differential expression of exosomal miRNAs between breast cancer patients with and without recurrence. Oncotarget. 2017 Jul 22;8(41).

8. Curtaz CJ, Reifschläger L, Strähle L, Feldheim J, Feldheim JJ, Schmitt C, et al. Analysis of microRNAs in Exosomes of Breast Cancer Patients in Search of Molecular Prognostic Factors in Brain Metastases. International Journal of Molecular Sciences 2022, Vol 23, Page 3683. 2022-03-27;23(7).

9. Fu L, Li Z, Zhu J, Wang P, Fan G, Dai Y, et al. Serum expression levels of microRNA-382‑3p, -598-3p, -1246 and -184 in breast cancer patients. Oncology Letters. 2016-07-01;12(1).

10. Jin X, Chen Y, Chen H, Fei S, Chen D, Cai X, et al. Evaluation of Tumor-Derived Exosomal miRNA as Potential Diagnostic Biomarkers for Early-Stage Non–Small Cell Lung Cancer Using Next-Generation Sequencing. Clinical Cancer Research. 2017/09/01;23(17).

11. Soliman SE-S, Abdelaleem AH, Alhanafy AM, Ibrahem RAL, Elhaded ASA, Assar MFA, et al. Circulating miR-21-5p and miR-126-3p: diagnostic, prognostic value, and multivariate analysis in non-small-cell lung cancer. Molecular Biology Reports 2021 48:3. 2021-04-10;48(3).
